# Plasma microRNA-221-3p as a biomarker for POCD after non-cardiac surgery

**DOI:** 10.1371/journal.pone.0275917

**Published:** 2022-10-11

**Authors:** Di Fan, Xuhui Chen, Hongli Zhou, Na Hu, Chengchuan Chen, Yi Yao, Yiping Bai, Jianguo Feng, Jing Jia, Xiaobin Wang

**Affiliations:** 1 Department of Anesthesiology, The Affiliated Hospital of Southwest Medical University, Luzhou, Sichuan Province, P. R. China; 2 Laboratoryof Anesthesiology, Southwest Medical University, Luzhou, Sichuan Province, P. R. China; Universita Politecnica delle Marche, ITALY

## Abstract

Our previous study showed that the plasma microRNA-221-3p level could serve as a biomarker for major depression or mood. This study aimed to further investigate the role of plasma microRNA-221-3p level in postoperative cognitive dysfunction (POCD). Patients undergoing non-cardiac surgery were randomly assigned according to the inclusion and exclusion criteria. POCD was diagnosed by the Z score method. The relative level of plasma microRNA-221-3p was decided by quantitative real-time polymerase chain reaction. Multiple logistic regression analysis and receiver operating characteristic(ROC) curves were used for the analysis of plasma microRNA-221-3p prediction performance for POCD. At 7 days post-surgery, the rate of POCD was 34.04%. Patients in the POCD group had a higher preoperative depression score, older age, and longer operation duration than that in the NPOCD group. The relative level of plasma microRNA-221-3p in the POCD group was 1.78 and 2.73 times higher than that in the NPOCD group at 1 day before and 7 days after the surgery, respectively. The relative content of plasma microRNA-221-3p at 7 days after operation was an independent risk factor for POCD. The ROC curves showed that the area under the curve was 0.938 for plasma microRNA-221-3p at postoperative 7 days, and the threshold for POCD detection was 12.33 with a sensitivity and specificity of 81.3% and 96.3%, respectively. Our results indicate that the plasma postoperative microRNA-221-3p levels could be an effective predictor for POCD after non-cardiac surgery.

## Introduction

Postoperative cognitive dysfunction (POCD) manifests as a poor postoperative cognition and severe limitations in intelligence, memory, and executive abilities [1]. POCD, which is a common complication, usually lasts for weeks or months in elderly patients who have undergone major surgeries [2]. Patients with POCD face a reduced self-care ability and quality of life, an increased demand for social concerns and medical support, and even an elevated mortality risk after surgery. However, the mechanism of POCD is not clear yet. Its diagnosis has only been based on clinical symptoms and a series of neuropsychological test scores, with no obvious imaging changes and atypical clinical manifestations. Therefore, a rapid, stable, and reliable biomarker for the diagnosis and evaluation of POCD should be urgently identified.

microRNAs are single-stranded, non-coding, small RNAs that play an important role in nervous system development, signal transduction, and tissue differentiation [3, 4]. As described in recent studies, microRNAs are associated with the development of neurological diseases, such as the following diseases of Alzheimer [5], Huntington [6], and Parkinson [7]. Owing to its clinical stability, sensitivity, and specificity, the peripheral blood contents of relevant microRNAs have become a biomarker of the corresponding diseases [8]. In our previous study, we found that perioperative depression was related to the plasma level of microRNA-221-3p [9]. Considering the close relationship between preoperative depression and POCD [10, 11], we speculate that microRNA-221-3p may be related to the occurrence and development of POCD. Therefore, the aim of this study was to investigate the correlation between plasma microRNA-221-3p and POCD, and to find further evidence for the prevention, clinical diagnosis, and efficacy evaluation of POCD.

## Materials and methods

### Patients

This is a single center randomized clinical study, in which patients undergoing non-cardiac surgery in the Affiliated Hospital of Southwest Medical University were randomly assigned according to the inclusion and exclusion criteria. The study was approved by the medical ethics committee of the affiliated hospital of Southwest Medical University(approval number: ky56294), all the individuals in this manuscript have been given written informed consent. The study was one of our team’s series researches on POCD, except for the different indicators in the blood, other methods were the same, so we used the previous clinical registration number(Trial registration: ChiCTR1800016435, registered 1 June 2018) [12, 13], To eliminate the role of learning memory for enhancing the credibility of the POCD diagnosis, 20 healthy volunteers were recruited as the control group in the study, and their cognitive function was only assessed.

**Fig 1:**
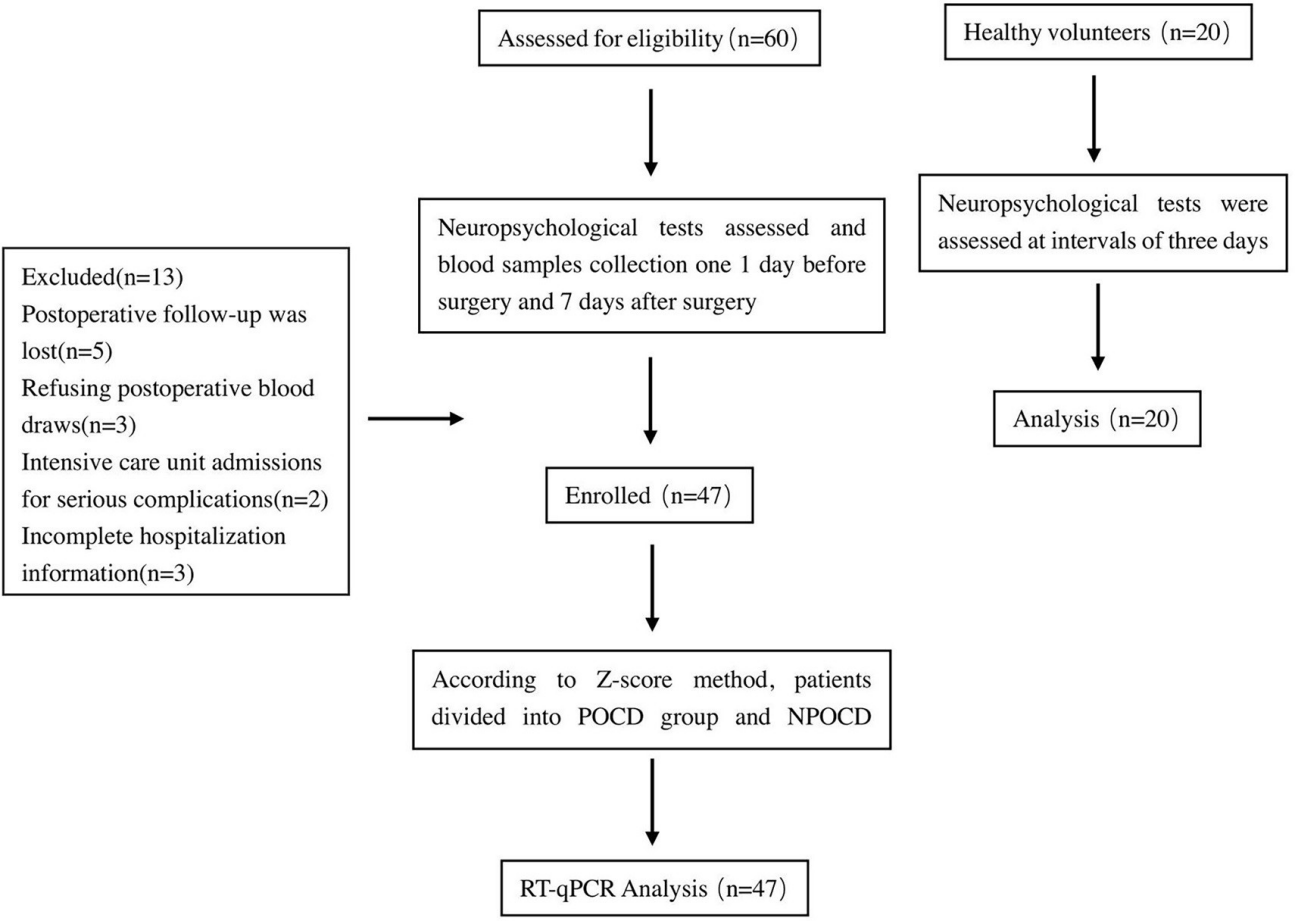
Study flow chart. RT-qPCR: quantitative real-time polymerase chain reaction; POCD: post-operative cognitive dysfunction.

The inclusion criteria were (1) American Society of Anesthesiologists (ASA) grade I–III; (2) age ≥ 45 years old; and (3) an expected hospital stay of at least 7days. The exclusion criteria were (1) a severe neuropsychiatric disease or craniocerebral trauma prior to the surgery; (2) drug dependence or alcohol addiction; (3) dementia or severe cognitive dysfunction prior to the surgery; (4) severe respiratory and cardiovascular complications; and (5) blood samples not meeting the test requirement. The experimental route is shown in Fig 1.

### Anesthesia

After the patients entered the operating room, the vital signs were monitored, the patients were given oxygen inhalation (> 5L/min). General anesthesia was induced with sufentanil (0.3–0.4ug/kg), propofol (1.5–2.0mg/kg) and CIS atracurium (0.2–0.3mg/kg). The narcotic and vasoactive drugs were used during the operation according to anesthesia depth, the blood pressure, and respiratory parameters. Dezocine 5mg and tropisetron 4.48mg were administered 30min prior to the end of the surgery, and a PCIA pump (sufentanate citrate 75–100ug, dezocine 10–15mg, and tropisetron 8.96–13.44mg) was connected after the surgery.

### Neuropsychological test evaluation and POCD assessment

One day before surgery, patients were assessed for depression using the patient health Questionnaire-9 (PHQ-9), while patients with previous severe cognitive impairment were excluded using the mini mental state examination (MMSE) (MMSE score <24). HKU-AHMU neuropsychological tests were performed 1 day before operation and 7 days after operation to evaluate the cognitive function of patients, including Chinese auditory learning test (CALT), digit span test (DST), judgment of line orientation test(JLOT) and language fluency test (VFT). All these tests were performed in a quiet environment by a trained doctor who was blinded to the surgical procedure and blood sample. At the same time, 20 healthy volunteers without surgery were recruited as the control group, with age, sex and education years matched. HKU-AHMU neuropsychological test was performed in control group to determine the normal reference value of cognitive function, with an interval of 7 days. Baseline scores and learning outcomes were subtracted from each neuropsychological test score according to the international guidelines for the study of postoperative cognitive impairment, and divided by the standard deviation of baseline scores in the control group. The result was designated a Z score. When at least two Z scales are greater than 1.961, POCD was considered.

### Blood sample collection

EDTA-K2 anticoagulant tubes were used to collect peripheral venous blood from the patients of preoperative 1 day and postoperative 7 days. Firstly, the blood sample was mixed upside down, and incubated for 30 min at room temperature. Secondly, was centrifuged for 15 min at 4°C 3000 rpm. Finally, the sample was placed in a medical cryogenic box at −80°C for analysis.

### Detection and preparation of plasma microRNA

Total RNA was extracted from plasma samples using TRIzol LS (Invitrogen, USA) according to the instructions from the manufacturer. Briefly, after homogenizing the plasma sample (250 μL) with TRIzol LS regent (750 μL), chloroform (200 μL) was added for further analysis. The sample was centrifuged at 12,000 × g, 4°C for 15 min, then the aqueous phase containing the RNA was transferred to a new tube and isopropanol (0.5 mL) was added for RNA precipitation. The RNA pellet was washed by 75% ethanol (1 mL), and finally resuspended in RNase-free water (10–20 μL), the RNA quality was determined with NanoDrop2000. An optical density ratio of 260/280 >1.80 was an indication that the sample could be used in subsequent experiments. Next, we used M-MLV reverse transcriptase (Promega, USA) for the synthesis of cDNA. A real-time quantitative polymerase chain reaction(qRT-PCR) was utilized for the final analysis according to the instructions from the SYBR Green PCR Master Mix Kit (Qiagen). The PCR parameters: 95°C for 10 min; 40 cycles at 95°C for 15 s, 60°C for 1 min, and 95°C for 15 s; and 60°C for 1 min and 95°C for 15s. microRNA-423-5p was chosen as a reference gene [9, 14, 15], fold change (2^−△△Ct^) was utilized to analyze plasma microRNA-221-3p relative levels. Related reverse transcription primers and PCR primers are shown in Tables 1 and 2, respectively.

**Table 1:** Specific reverse transcription primers of microRNA-423-5p and microRNA-221-3p.

Gene	Primer sequence	Primer length (bp)
hsa-microRNA-423-5p-RT	5’GTCGTATCCAGTGCGTGTCGTGGAGTCGGCAATTGCACTGGATACGACAAAGTCT3’	58
hsa-microRNA-221-3p-RT	5’GTCGTATCCAGTGCGTGTCGTGGAGTCGGCAATTGCACTGGATACGACGAAACCC3’	55

**Table 2:** The primers and sequences for detecting genes by qRT-PCR.

Gene	Primer sequence	Primer length (bp)
hsa-microRNA-qR (common post primer of microRNA-423-5p and microRNA-221-3p)	CAGTGCGTGTCGTGGAGT	18
hsa-microRNA-423-5p-qF	TGAGGGGCAGAGAGCG	16
hsa-microRNA-221-3p-qF	GGGAAGCTACATTGTCTGC	19

### Sample size calculation

In our previous study, the level of the PHQ-9 scores and serum miR-221-3p Pearson’s correlation coefficient is 0.506 [9]. In the study, with 80% power and a 5% level of significance, the calculated sample size included a 10% dropout rate, requiring a total of 41 participants.

### Statistical analysis

Kolmogorov-Smirnov test was used to assess the normal distribution of continuous data. Normally distributed continuous data were expressed as mean ± standard deviation(SD) and were analyzed utilizing Student’s t-test. Categorical data were expressed as percentages or frequencies and analyzed using Fisher’s exact probability test or Pearson’s chi-square test. The independent predictors of POCD were analyzed by binary logistic regression, and the results were expressed as odds ratios(ORs). The prediction performance of microRNA-221-3p for POCD was measured using subject operating characteristics, and the results were determined by the 95% confidence intervals and area under the curve. P<0.05 was considered to be statistically significant.

## Results

### The baseline values from the control and operation groups

**Fig 2:**
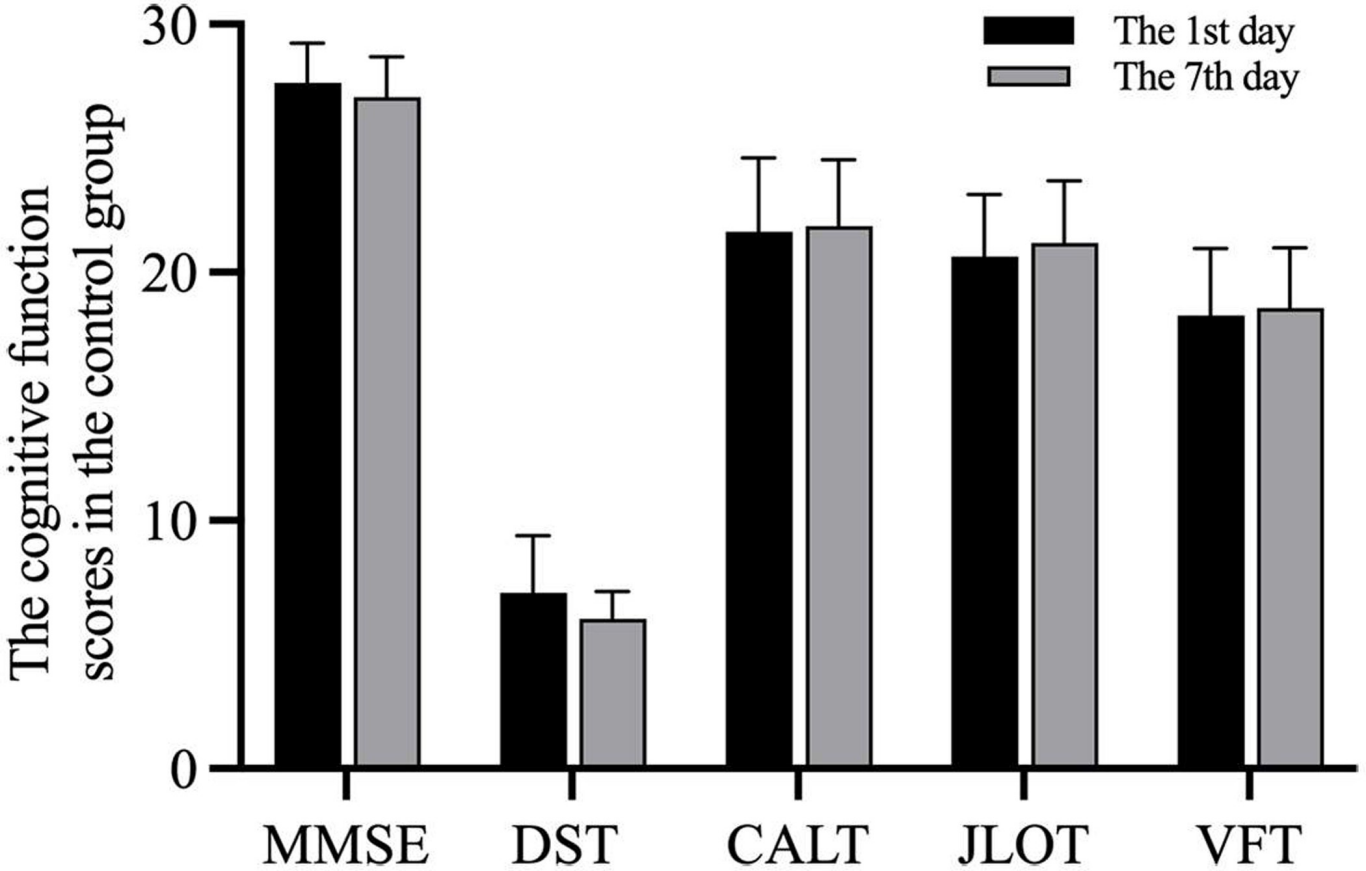
The cognitive function scores from the control group. MMSE: Mini-Mental State Examination; CALT: Chinese Auditory Learning Test; DST: Digital Span Test; VFT: Verbal Fluency Test; JLOT: Judgment of Line Orientation Test.

A total 60 patients with non-cardiac surgeries were considered for inclusion in the study. Among them, 13 were excluded (5 patients due to postoperative follow-up, 3 patients for refusing postoperative blood draws, 2 patients due to intensive care unit admissions for serious complications, and 3 patients for incomplete hospitalization information). Finally, 47 patients with non-cardiac surgeries were included in this study. In addition, 20 healthy volunteers with no operation were selected as a control group, there were no statistically significant differences in the basic data between the control and operated groups (Table 3, P>0.05). There were also no statistically significant differences in the cognitive function of the control group at intervals of 7 days (Fig 2, P > 0.05).

**Table 3:** The general data and basic cognitive functions of participants from control and operation groups.

Variable	The first data in the control group (n = 20)	The preoperative data in the operation group (n = 47)	*P-*value
Age (years)	64.90±9.85	63.19±9.22	0.499
Gender (male/female)	13/7	26/21	0.462
Years of education	6.15±4.18	5.23±3.62	0.369
Marital status			0.170
Single	14	39	
Married	1	3	
Divorced	3	5	
Widowed	2	0	
Baseline PHQ-9 scores	7.80±4.07	8.81±5.22	0.445
Baseline MMSE scores	27.65±1.56	27.26±1.68	0.380
Baseline DST scores	7.10±2.29	7.29±1.85	0.711
Baseline CALT scores	21.65±2.96	22.12±2.78	0.530
Baseline JLOT scores	20.65±2.50	21.34±2.64	0.323
Baseline VFT scores	18.65±2.60	19.89±2.73	0.088

Values are presented as mean ± standard deviation (SD). MMSE: Mini-Mental State Examination; PHQ-9: Patient Health Questionaire-9; CALT: Chinese Auditory Learning Test; DST: Digital Span Test; VFT: Verbal Fluency Test; J LOT: Judgment of Line Orientation Test.

### The cognitive function scores from the NPOCD groups and POCD

**Fig 3:**
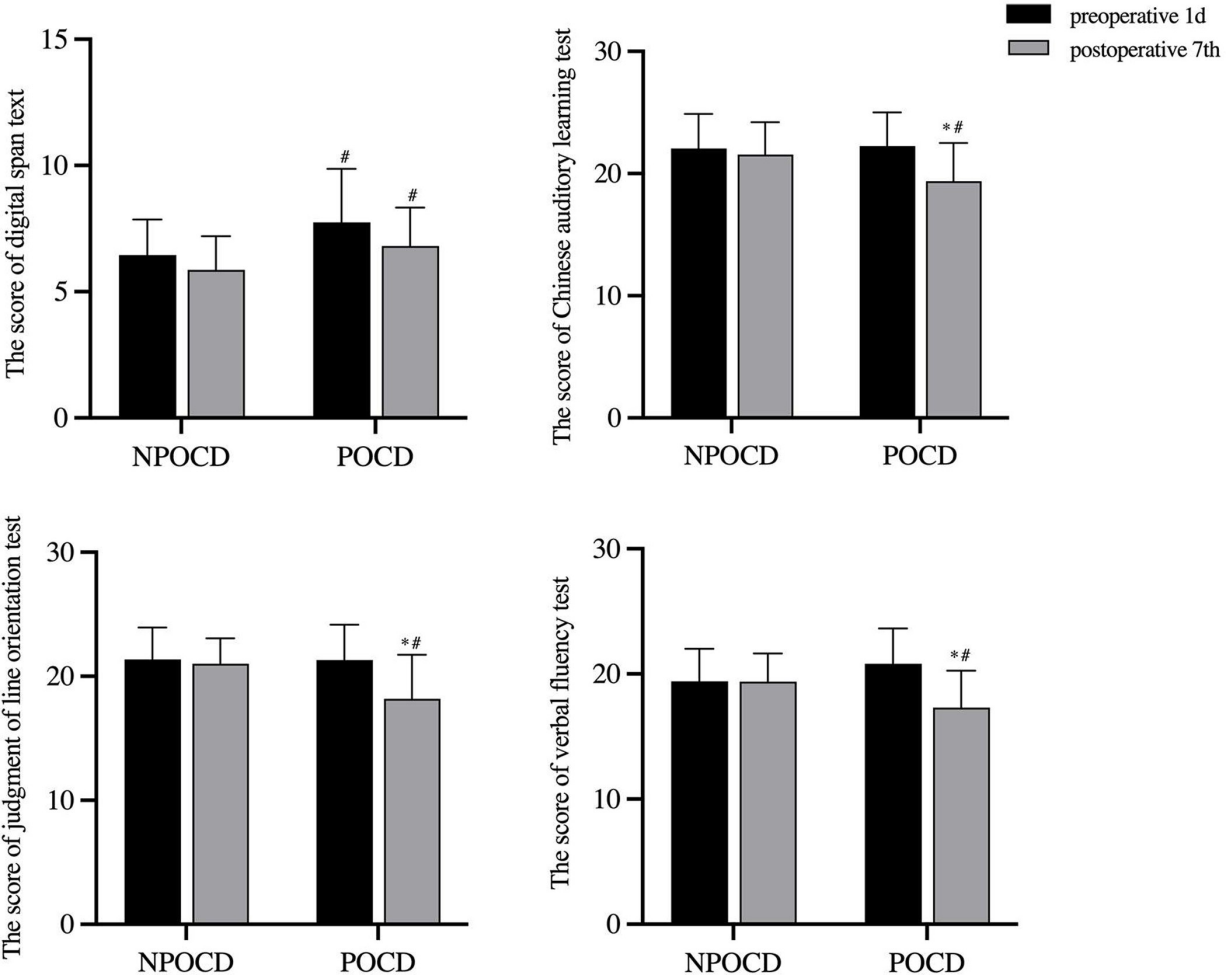
The neuropsychological test scores from NPOCD and POCD group. Vs 1 day before surgery, *p < 0.05, vs the NPOCD group, #p < 0.05.

As shown in Fig 3, compared with the NPOCD group, the DST of the POCD group were increased at 1 day before the surgery(p = 0.017, t = 2.475) and at 7 days after the surgery (P = 0.034, t = 2.188), while the reduction in the CALT, JLOT and VFT scores was seen at 7 days after the surgery in the POCD(p = 0.016, t = -2.505; p = 0.001, t = -3.445; p = 0.010, t = -2.688). Compared with 1 day before the surgery in the POCD group, there were lower CALT, JLOT and VET scores (p < 0.001, t = 6. 446; p < 0.001, t = 4.592; p < 0.001, t = 5.780) at 7 days in the POCD group.

### The general data from the operated patients

As shown in Table 4, a total of 16 patients developed POCD with an incidence of 34.04% according to the International Study of Postoperative Cognitive Dysfunction criterion. Compared with the NPOCD group, the POCD group included statistically significantly older patients, with longer operation duration, and a higher preoperative depression scores (P < 0.001, t = 5.003; P < 0.001, t = 4.696; p = 0.016, t = 2.493). However, there were no differences between the two groups with regard to gender, years of education, the surgery type, an American Society of Anesthesiology(ASA) grade, fluid input, blood loss, and a preoperative MMSE score (P>0.05).

**Table 4:** The general characteristics of NPOCD and POCD groups.

Variable	NPOCD group (n = 31)	POCD group (n = 16)	t/χ2 value	*P*-value
Age (years)	58.94±5.85	71.43±9.06	5.003	<0.001*
Gender (male/female)	17/14	9/7	0.009	0.927
Years of education (years)	5.61±3.77	6.19±3.87	0.49	0.626
Surgery type				
Gastrointestinal surgery	15	5	5.312	0.150
Arthroscopic surgery	8	3		
Gynecologic surgery	3	6		
Thoracic surgery	5	2		
ASA classification (I/II/III)	4/16/11	2/7/7	0.209	0.647
Operation duration (min)	125.65±43.58	195.63±56.86	4.696	<0.001*
Intraoperative fluid input (ml)	1679.03±438.90	1587.50±491.09	0.651	0.519
Intraoperative blood loss (ml)	312.90±129.72	378.13±177.92	1.436	0.158
Preoperative PHQ-9 scores	7.52±4.66	11.31±5.47	2.493	0.016*
Preoperative MMSE scores	27.58±1.63	26.63±1.71	0.761	0.451

Values are presented as mean ± standard deviation (SD). PHQ-9: Patient Health Questionaire-9; MMSE: Mini-Mental State Examination; ASA: American Society of Anesthesiology. Vs the NPOCD group,

*p < 0.05.

### The plasma microRNA-221-3p relative levels between the NPOCD and POCD groups

**Fig 4:**
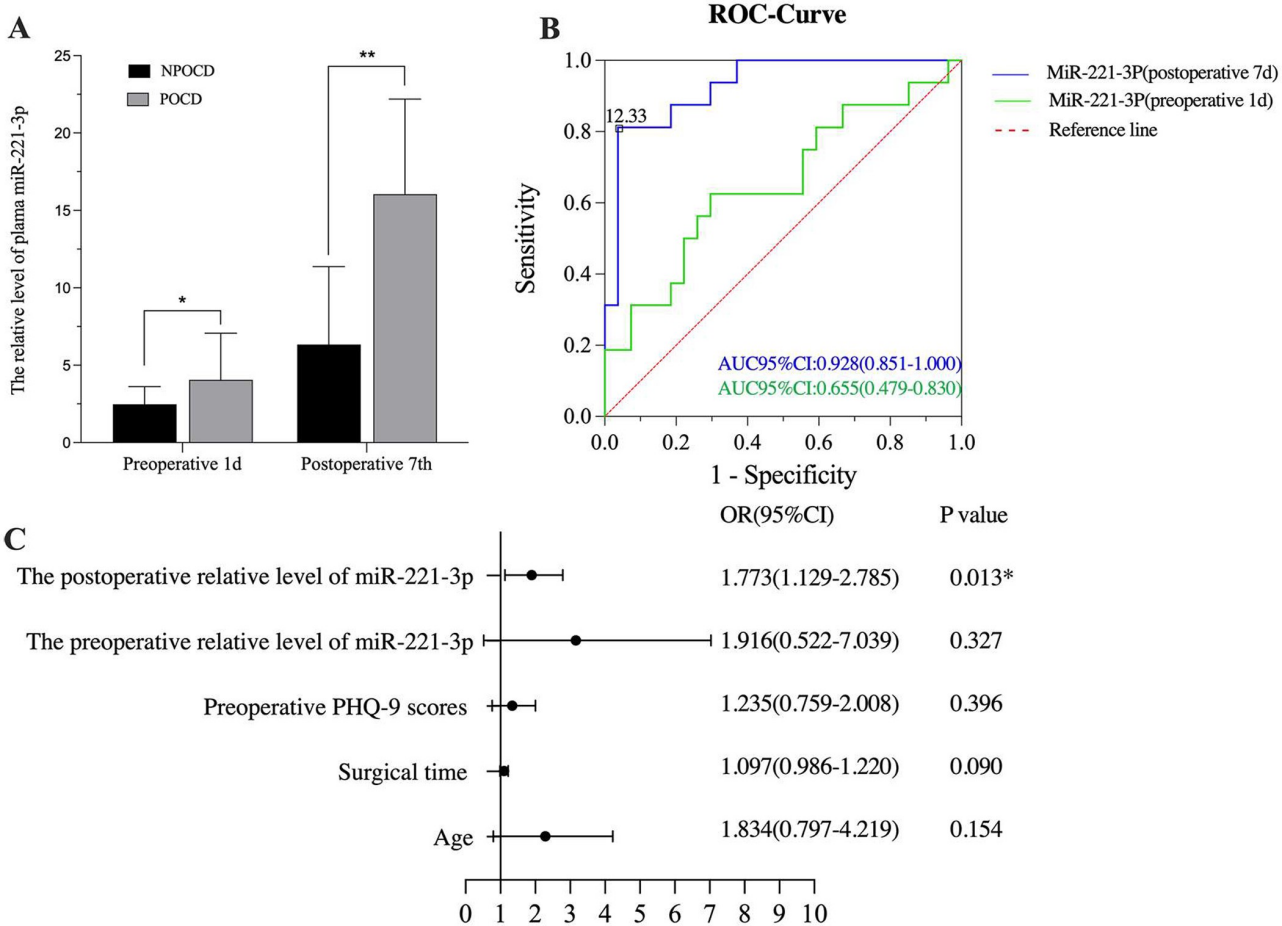
The relationship between plasma microRNA-221-3p relative levels and POCD. A. The relative levels of microRNA-221-3p of NPOCD and POCD groups from q-RT-PCR. Vs the NPOCD group in preoperative 1d, *p < 0.05, vs the NPOCD group in postoperative 7d, **p < 0.01. B. The results from binary logistic regression analysis. OR: odds ratio, CI: confidence interval, *p < 0.05. C. ROC analysis of the plasma miR-221-3p relative levels. AUC: Area Under Curve, ROC: Receiver operating characteristic.

As shown in Fig 4A, there was a significant difference in the relative plasma microRNA-221-3p levels at the preoperative 1 day (t = 2.779, P = 0.008) and postoperative 7 days (t = 4.711, P < 0.0001) between the NPOCD and POCD groups, but the binary logistic regression analysis found that the plasma microRNA-221-3p relative level of the postoperative 7 days was only independently associated with POCD (Fig 4B, OR:0.715, 95% CI:0.520–0.912). The receiver operating characteristic (ROC) curves showed the predictive performance of plasma microRNA-221-3p for the development of POCD (Fig 4C). The areas under the curve for plasma microRNA-221-3p levels were 0.682 (95% CI:0.4794 to 0.8308, P < 0.05) and 0.938(95% CI:0.8513–1.000, P < 0.01) on the preoperative 1 day and postoperative 7 days, suggesting that the plasma microRNA-221-3p level on the postoperative 7 days could be strongly predictive for POCD. The threshold for POCD was 12.33 with a sensitivity and specificity of 81.3% and 96.3%, respectively.

## Discussion

POCD is defined as a postoperative impairment of the memory, attention, logical thinking ability, and orientation accompanied by a decline in self-care and social skills. POCD differs from delirium in that the latter is a reversible acute overall mental disorder that may be followed by an altered level of consciousness [16]. More recently, it has been recommended to describe all of the perioperative cognitive changes that occur before and 12 months after a surgery as perioperative neurocognitive disorders (PND) [17]. Since this study mainly focused on the cognitive decline of patients during the early postoperative period, the term "POCD" was still used in our study. Due to the low specificity and sensitivity of the MMSE scale and the high effectiveness of its clinical application, the MMSE scale was used to remove the patients with preoperative severe cognitive dysfuction (MMSE score <24). Finally, we chose the HKU-AHMU neuropsychological tests to determine patients’ cognitive function in terms of language, memory, vision, and spatial awareness. During the neuropsychological test, we ensured the test completion within 30 minutes and chose to perform the tests at the patients’ bedside in the evening after dinner. Considering that perioperative anesthetic drugs and pain may affect patients’ cognitive function, we chose to complete the tests one day before and seven days after the surgery, which was in accordance with the majority of previous research [18, 19]. In order to circumvent the learning effect, 20 volunteers matched to the operation group for age, gender, education, and baseline neuropsychological test scores were included in the study as a control group and were neuropsychologically tested 7 days apart, and there were no significant differences at the interval of 7 days in the control group.

All of the enrolled patients were assessed using the HKU-AHMU neuropsychological tests, and POCD was diagnosed when the Z value of at least two scales was greater than or equal to 1.96 [1]. In the study, the incidence of POCD was 34.04%, compared with 5%–55% in patients undergoing other major operations [1, 20, 21]. These different results may be due to the surgery types, anesthetic methods, diagnostic tools, and testing times. A higher incidence of POCD has been reported after cardiac surgeries [22]. However, given the sample size and trial feasibility, we only included patients undergoing non-cardiac surgeries, and this sample insufficiency may be a limitation. As shown in our study, preoperative DST scores were higher in the POCD group than that in the NPOCD group, possibly due to the ceiling effect [23], where patients already have a "cognitive reserve," and a cognitive decline may be undetected or underestimated. In contrast, the POCD group had lower CALT, JLOT and VFT scores 7 days after the surgery, indicating that patients with POCD mainly present with language and memory dysfunction after surgery.

In our study, the age and duration of surgery were related to POCD, which may be caused by the degeneration of brain tissue, slowing of liver and kidney function, and accumulation of anesthetic drugs in elderly patients [13]. However, these factors were not independent predictors of POCD from the multiple logistic regression analysis and the reasons may have been related to differences in the types of surgery, sample size, and age of the participants. In addition, preoperative depression was also closely related to POCD. Several studies have confirmed that preoperative depression may contribute to the development of POCD, possibly due to hippocampal atrophy, reduced brain-derived neurotrophic factor expression, and increased production of inflammatory cytokines [10, 11].

microRNAs are non-coding small RNAs that can regulate and target various proteins, genes, and signaling pathways [3, 4]. Owing to their stable expression and ease of detection, blood microRNAs have been widely used as biomarkers for the diagnosis and prognosis of many diseases [5, 9, 24, 25]. In the study, plasma microRNA-221-3p levels were higher in the POCD group, and a subsequent binary regression analysis found that the postoperative plasma microRNA-221-3p level was an independent predictor of POCD. Most importantly, we established ROC curves for plasma microRNA-221-3p levels, and the results showed excellent performance for POCD identification. Previous studies have reported that microRNA-221-3p is related to the pathogenesis of many cerebrovascular diseases, the microRNA-221-3p levels in plasma and CSF can be used as risk and diagnostic indicators of PSD (poststroke depression), moreover, the upregulated plasma microRNA-133b and microRNA-221-3p levels may be biomarkers for early PD [26]. In addition, microRNA-221-3p plays a role in the autophagy of dopaminergic neurons in a mouse model of PD [27]. Notably, there are certain commonalities in the occurrence of cerebrovascular disease and POCD. Therefore, these results support that the plasma expression levels of microRNA-221-3p are significantly increased in patients with POCD, suggesting a potential contribution of microRNA-221-3p in patients with POCD. In this study, age, preoperative PHQ-9 scores, and preoperative plasma miR-221-3p level were significantly different between the NPOCD and POCD groups, but the binary logistic regression found that age, preoperative PHQ scores, surgery duration and preoperative plasma microRNA-221-3p content were not independent risk factors for POCD. We regreted that we did not observe whether age and surgery duration would affect the change of plasma microRNA-221-3p content, which needs to be further clarified in future studies. However, this observation found that high postoperative plasma microRNA-221-3p level was independent risk factors for POCD, the mechanism may be related to its proinflammatory effect. With regards to the regulatory mechanism of microRNA-221-3p in related diseases, Wang et al. found that microRNA-221-3p ameliorated sevoflurane-induced cell injury by promoting cellular activity and inhibiting apoptosis through suppression of CDKN1B expression [28]. Numerous studies implicated a relationship between microR-221-3p and inflammation, microRNA-221-3p could mediate the antitumor effects of IFN-α [29], microRNA-221-3p and microRNA-92a-3p are reportedly associated with smoking-induced inflammation in COPD (chronic obstructive pulmonary disease) [30], microRNA-221-3p could drive the shift of macrophages towards pro-inflammatory functions by inhibiting the activation of JAK3/STAT3 [31]. Moreover, Meng et al. found that plasma microRNA-221-3p was positively correlated with the expression levels of TNF-α, IL-17A, and IL-22 in patients with psoriasis [24]. Presently, there are several hypotheses about the pathogenesis of POCD, which are related to inflammation of the central nervous system, nerve cell apoptosis, dysfunction of the cholinergic nervous system, and oxidative stress injury. However, inflammation of the nervous system still plays an important role in the development of POCD [16, 32, 33]. According to our previous study, microRNA-221-3p could target IRF2 of astrocytes to increase IFN-α expression and regulate nervous system inflammation [9]. We suggest that plasma microRNA-221-3p level plays an important role in inflammation, leading to a local and systematic inflammatory response and causing synaptic plasticity damage, neuronal degeneration and death, and synaptic related protein reduction.

## Conclusions

In summary, our results indicate that the plasma postoperative microRNA-221-3p relative levels could be an effective predictor for POCD after non-cardiac surgery, the mechanism may be related to the involvement of microRNA-221-3p in inflammatory response. However, there are some disadvantages on the study. First, all of the samples were from a single center in China, further studies with large samples from multiple centers and basic research are needed. Second, the main purpose was to observe whether the higher preoperative depression score was more prone to POCD, so we did not consider the post-operative depression score, it would be interesting to see how the depressive state of the patients is impacted by a non-cardiac surgical procedure, and whether an increase in depression scores post-surgery correlate with higher levels of miR-221-3p, therefore, we will make it clear in future research. Third, we are planning to assess microRNA-221-3p levels and proinflammatory markers in cerebrospinal fluid for investigating whether these correlate with higher expression of miR-221-3p and POCD.

## References

[1] Moller JT, Cluitmans P, Rasmussen LS, Houx P, Rasmussen H, Canet J, et al. Long-term postoperative cognitive dysfunction in the elderly ISPOCD1 study. ISPOCD investigators. International Study of Post-Operative Cognitive Dysfunction. Lancet. 1998; 351(9106): 857–861. doi: 10.1016/s0140-6736(97)07382-0 . 9525362

[2] Daiello LA, Racine AM, Yun Gou R, Marcantonio ER, Xie Z, Kunze LJ, et al. Postoperative delirium and postoperative cognitive dysfunction: overlap and divergence. Anesthesiology. 2019; 131(3): 477–491. doi: 10.1097/aln.0000000000002729 . 31166241 PMC6692220

[3] Murchison EP, Hannon GJ. miRNAs on the move: miRNA biogenesis and the RNAi machinery. Curr Opin Cell Biol. 2004; 16(3): 223–229. doi: 10.1016/j.ceb.2004.04.003 . 15145345

[4] Wei C, Luo T, Zou S, Zhou X, Shen W, Ji X, et al. Differentially expressed lncRNAs and miRNAs with associated ceRNA networks in aged mice with postoperative cognitive dysfunction. Oncotarget. 2017; 8(34): 55901–55914. doi: 10.18632/oncotarget.18362 . 28915561 PMC5593532

[5] Swarbrick S, Wragg N, Ghosh S, Stolzing A. Systematic review of miRNA as biomarkers in Alzheimer’s disease. Mol Neurobiol. 2019; 56(9): 6156–6167. doi: 10.1007/s12035-019-1500-y . 30734227 PMC6682547

[6] Tung CW, Huang PY, Chan SC, Cheng PH, Yang SH. The regulatory roles of microRNAs toward pathogenesis and treatments in Huntington’s disease. J Biomed Sci. 2021; 28(1): 59. doi: 10.1186/s12929-021-00755-1 . 34412645 PMC8375176

[7] Kuo MC, Liu SC, Hsu YF, Wu RM. The role of noncoding RNAs in Parkinson’s disease: biomarkers and associations with pathogenic pathways. J Biomed Sci. 2021; 28(1): 78. doi: 10.1186/s12929-021-00775-x . 34794432 PMC8603508

[8] Benes V, Castoldi M. Expression profiling of microRNA using real-time quantitative PCR, how to use it and what is available. Methods. 2010; 50(4): 244–249. doi: 10.1016/j.ymeth.2010.01.026 . 20109550

[9] Feng J, Wang M, Li M, Yang J, Jia J, Liu L, et al. Serum miR-221-3p as a new potential biomarker for depressed mood in perioperative patients. Brain Res. 2019; 1720: 146296. doi: 10.1016/j.brainres.2019.06.015 . 31211948

[10] Patron E, Messerotti Benvenuti S, Zanatta P, Polesel E, Palomba D. Preexisting depressive symptoms are associated with long-term cognitive decline in patients after cardiac surgery. Gen Hosp Psychiatry. 2013; 35(5): 472–479. doi: 10.1016/j.genhosppsych.2013.05.004 . 23790681

[11] Oldham MA, Lin IH, Hawkins KA, Li FY, Yuh DD, Lee HB. Depression predicts cognitive and functional decline one month after coronary artery bypass graft surgery (Neuropsychiatric Outcomes After Heart Surgery study). Int J Geriatr Psychiatry. 2021; 36(3): 452–460. doi: 10.1002/gps.5443 . 33022808 PMC9326959

[12] Wang M, Su P, Liu Y, Zhang X, Yan J, An X, et al. Abnormal expression of circRNA_089763 in the plasma exosomes of patients with postoperative cognitive dysfunction after coronary artery bypassgrafting. Molecular Medicine Reports. 2019; 20: 2549–2562. doi: 10.3892/mmr.2019.10521 . 31524256 PMC6691254

[13] Zhou H, Li F, Ye W, Wang M, Zhou X, Feng J, et al. Correlation between plasma circRNA-089763 and postoperative cognitive dysfunction in elderly patients undergoing non-cardiac surgery. Front Behav Neurosci. 2020; 14: 587715. doi: 10.3389/fnbeh.2020.587715 . 33132863 PMC7573279

[14] Liu X, Zhang L, Cheng K, Wang X, Ren G, Xie P. Identification of suitable plasma-based reference genes for miRNAome analysis of major depressive disorder. J Affect Disord. 2014; 163: 133–139. doi: 10.1016/j.jad.2013.12.035 . 24479999

[15] Wan Y, Liu Y, Wang X, Wu J, Liu K, Zhou J, et al. Identification of differential microRNAs in cerebrospinal fluid and serum of patients with major depressive disorder. PLoS One. 2015; 10(3): e0121975. doi: 10.1371/journal.pone.0121975 . 25763923 PMC4357380

[16] Vlisides P, Avidan M. Recent Advances in Preventing and Managing Postoperative delirium. F1000Res. 2019; 8. doi: 10.12688/f1000research.16780.1 . 31105934 PMC6498743

[17] Evered L, Silbert B, Knopman DS, Scott DA, DeKosky ST, Rasmussen LS, et al. Recommendations for the Nomenclature of Cognitive Change Associated with Anaesthesia and Surgery-2018. Anesthesiology. 2018; 129(5): 872–879. doi: 10.1097/aln.0000000000002334 . 30325806

[18] Duan X, Zhu T, Chen C, Zhang G, Zhang J, Wang L, et al. Serum glial cell line-derived neurotrophic factor levels and postoperative cognitive dysfunction after surgery for rheumatic heart disease. J Thorac Cardiovasc Surg. 2018; 155(3): 958-965.e951. doi: 10.1016/j.jtcvs.2017.07.073 . 28918204

[19] Wu C, Wang R, Li X, Chen J. Preoperative serum microRNA-155 expression independently predicts postoperative cognitive dysfunction after laparoscopic surgery for colon cancer. Med Sci Monit. 2016; 22: 4503–4508. doi: 10.12659/msm.898397 . 27872469 PMC5123778

[20] Monk TG, Weldon BC, Garvan CW, Dede DE, van der Aa MT, Heilman KM, et al. Predictors of cognitive dysfunction after major noncardiac surgery. Anesthesiology. 2008; 108(1): 18–30. doi: 10.1097/01.anes.0000296071.19434.1e . 18156878

[21] McDonagh DL, Mathew JP, White WD, Phillips-Bute B, Laskowitz DT, Podgoreanu MV, et al. Cognitive function after major noncardiac surgery, apolipoprotein E4 genotype, and biomarkers of brain injury. Anesthesiology. 2010; 112(4): 852–859. doi: 10.1097/ALN.0b013e3181d31fd7 . 20216394 PMC2933423

[22] van Harten AE, Scheeren TW, Absalom AR. A review of postoperative cognitive dysfunction and neuroinflammation associated with cardiac surgery and anaesthesia. Anaesthesia. 2012; 67(3): 280–293. doi: 10.1111/j.1365-2044.2011.07008.x . 22321085

[23] Funder KS, Steinmetz J, Rasmussen LS. Methodological issues of postoperative cognitive dysfunction research. Semin Cardiothorac Vasc Anesth. 2010; 14(2): 119–122. doi: 10.1177/1089253210371520 . 20478952

[24] Meng Z, Qiu J, Zhang H. MiR-221-3p as a Potential Biomarker for patients with psoriasis and its role in inflammatory responses in keratinocytes. Skin Pharmacol Physiol. 2021; 34(5): 300–306. doi: 10.1159/000515114 . 34091460

[25] Chen Q, Deng N, Lu K, Liao Q, Long X, Gou D, et al. Elevated plasma miR-133b and miR-221-3p as biomarkers for early Parkinson’s disease. Sci Rep. 2021; 11(1): 15268. doi: 10.1038/s41598-021-94734-z . 34315950 PMC8316346

[26] Cui Y, Ma G, Kong F, Song L. Diagnostic values of miR-221-3p in serum and cerebrospinal fluid for post-stroke depression and analysis of risk factors. Iran J Public Health. 2021; 50(6): 1241–1249. doi: 10.18502/ijph.v50i6.6423 . 34540745 PMC8410966

[27] Lang Y, Li Y, Yu H, Lin L, Chen X, Wang S, et al. HOTAIR drives autophagy in midbrain dopaminergic neurons in the substantia nigra compacta in a mouse model of Parkinson’s disease by elevating NPTX2 via miR-221-3p binding. Aging (Albany NY). 2020; 12(9): 7660–7678. doi: 10.18632/aging.103028 . 32396526 PMC7244061

[28] Wang Q, Tian X, Lu Q, Liu K, Gong J. Study on the ameliorating effect of miR-221-3p on the nerve cells injury induced by sevoflurane. Int J Neurosci. 2022; 132(2): 181–191. doi: 10.1080/00207454.2020.1806267 . 32900248

[29] Kneitz B, Krebs M, Kalogirou C, Schubert M, Joniau S, van Poppel H, et al. Survival in patients with high-risk prostate cancer is predicted by miR-221, which regulates proliferation, apoptosis, and invasion of prostate cancer cells by inhibiting IRF2 and SOCS3. Cancer Res. 2014; 74(9): 2591–2603. doi: 10.1158/0008-5472.Can-13-1606 . 24607843

[30] Shen Y, Lu H, Song G. MiR-221-3p and miR-92a-3p enhances smoking-induced inflammation in COPD. J Clin Lab Anal. 2021; 35(7): e23857. doi: 10.1002/jcla.23857 . 34097306 PMC8274981

[31] Quero L, Tiaden AN, Hanser E, Roux J, Laski A, Hall J, et al. miR-221-3p drives the shift of M2-macrophages to a pro-inflammatory function by suppressing JAK3/STAT3 activation. Front Immunol. 2019; 10: 3087. doi: 10.3389/fimmu.2019.03087 . 32047494 PMC6996464

[32] Li Z, Zhu Y, Kang Y, Qin S, Chai J. Neuroinflammation as the underlying mechanism of postoperative cognitive dysfunction and therapeutic strategies. Front Cell Neurosci. 2022; 16: 843069. doi: 10.3389/fncel.2022.843069 . 35418837 PMC8995749

[33] Tan XX, Qiu LL, Sun J. Research Progress on the role of inflammatory mechanisms in the development of postoperative cognitive dysfunction. Biomed Res Int. 2021; 2021: 3883204. doi: 10.1155/2021/3883204 . 34869762 PMC8642009

